# Building reparative futures demands a reckoning with the crisis form of global health

**DOI:** 10.1371/journal.pgph.0006473

**Published:** 2026-05-20

**Authors:** Sharifah Sekalala, Shajoe Lake

**Affiliations:** Centre for Global Health Law, School of Law, University of Warwick, Coventry, United Kingdom; Harvard Medical School, UNITED STATES OF AMERICA

## Abstract

Recent cuts to global health funding and US withdrawal from the World Health Organization have generated claims that global health has entered a new crisis. This essay argues that such accounts misread the present moment. The better view is that global health operates through a ‘crisis form’, a modality of governance in which structural inequality is treated as ordinary, acute disruptions (Big Events) are framed as emergencies, and responses to Big Events restore institutional authority without transforming the historically continuous racialized political economy in which global health is embedded. Drawing on past Big Events we trace this pattern from colonial medicine, through Alma Ata, HIV/AIDS, Ebola to COVID-19, showing how each Big Event exposed the underlying structural problems in global health and generated promises of change while preserving structural inequality. We then analyze early 2025 commentaries on aid cuts and US withdrawal from the World Health Organization to show how crisis discourse, even at a moment ripe with decolonial talk, channels political energy into the language of rescue and renewal, leaving deeper systemic questions of redress and redistribution untouched. In response, the essay develops reparatory global health as a political and conceptual orientation that calls for a refusal of restoration by taking the structural problems exposed by Big Events as the object of action, and by treating reparations as transformative, tactical, and oriented towards structural change and non-recurrence.

## Crises talk and the foreclosure of repair

The Trump’s administrations 2025 decision to withdraw from the World Health Organization (WHO) and cut 83% of its USAID-funded programming triggered a familiar response across the global health system, with commentators warning that global health has entered another crisis. This retrenchment coincides with similar, if less visible, funding cuts across Europe: Belgium (25%), the Netherlands (30%), France (37%), the UK (40%) [1,2]. Yet what is being narrated as a breakdown through these recent ‘Big Events’ has made visible the racialized political economy in which some populations remain systematically vulnerable to preventable death while others retain the power to define what this moment means and determine the terms of response and recovery.

For the global majority, this condition is not exceptional. Global health has perennially been characterized by a perpetual state of crisis. Communities across the Global South have faced outbreaks that never rise to the threshold of global alarms, chronic funding insecurity because donor priorities do not align with local needs, and intensifying health harms linked to climate change, debt and weak public health systems [3–6]. This was exceptionally visible throughout the COVID-19 pandemic. Millions of Black and brown people died while predominantly white, wealthy nations hoarded vaccines, defended patent monopolies and blocked efforts to expand manufacturing in the Global South [7,8]. Contra claims that this was a departure from the ordinary logic of global health, the better view is that this was an acute expression of a longer history in which colonial medicine treated racialized peoples as expendable subjects of experimentation while locating scientific authority and pharmaceutical control elsewhere. That same logic persists in outsourcing of clinical trials, the interpretation and enforcement of WTO intellectual property (IP) rules, and the continued restriction of local pharmaceutical production across Africa and other parts of the Global South [8–11].

We use the concept of the ‘crisis from of global health’ to make sense of this condition. Foreign aid became necessary only after colonialism and imperialism made it necessary. Global health remains embedded in that order. Some countries are made to carry heavier disease burdens, weaker health systems, more debt, less fiscal space, and less authority within the institutions that govern them, while others retain the power to define priorities and monopolize the control of resources and terms of repair. These inequalities are grounded in the material and epistemic organization of global health itself and through the wider racialized global political economy of which it is constitutive [12,13]. The crisis form names a pattern in which enduring structural instability is treated as normal, while acute disruptions are selectively framed as crises. Communities forced to survive funding shocks, epidemics, debt burdens, and institutional abandonment, rarely secure the time, resources and political space needed for repair. Thus, crisis talk organizes a politics of postponement in which structural redress is deferred in the name of urgency, resilience, and emergency response.

This essay takes reparatory global health as its political and conceptual standpoint. We begin with the recognition that present health inequalities were historically produced. Imperialism and coloniality, across racial slavery, colonialism, neo-colonialism and neo-liberal restructuring, have shaped the very conditions in which global health emerged and continues to operate. Debt, IP monopolies, ecological extraction and epistemic domination are the constitutive conditions of global health. Thinking about crises in global health is therefore misleading when it begins with the assumption that a fundamental legitimate system has failed to distribute care fairly. A more adequate starting point recognizes that harm has been built into its historical formation. From that perspective, reparatory global health asks what redress is owed when present inequalities are historically accumulated, institutionally produced, and politically maintained. It is orientated toward redress, transformation and non-recurrence. It asks not only how to compensate those who have been harmed, but how to dismantle and remake the arrangements that keep reproducing harm in new forms.

That starting point also changes how we read the present. For communities in the Global South and marginalized groups in the Global North, this terrain is familiar. Many have long built infrastructures of care, resistance and solidarity beyond western validation or leadership, and have not only endured abandonment but also sustained other ways of organizing health and life. Drawing from those struggles, from Alma-Ata to global coalitions and theories of reparative world-making [14,15], we argue for a horizon of global health grounded in mutuality, redistribution and epistemic plurality. The present moment should therefore be read less as a collapse of a previously stable order than as an opening to reconfigure the terms of global cooperation by centering those who have long been subjected to the foreign gaze [11].

## A historical account of the crisis form of global health

The concept of the crisis form of global health becomes clearer when read historically. First, if we take seriously that the ‘global’ in global health inherits international (and world health due to the incorporation of world systems theory), then ‘global’ becomes an analytic for the specific historical relations constitutive of colonial medicine to our present world, not simply a descriptor for a ‘loosely unified set of institutions, discourses and practices,’ which go beyond the nation state as the primary object of analysis [16]. Second, given the form and content of global health, it ought to be appreciated that global health has been organized through a recurring pattern in which structural inequality is normalized, acute disruptions are rendered visible as Big Events or emergencies, and the response to those emergencies restores authority without disturbing the order that produced harm. Here, we distinguish between ‘enduring crisis’ and ‘heightened visibility’. Enduring crisis refers to the ordinary organization of the global health order, in which some populations live with weak health systems, debt burdens, fragile infrastructures, uneven exposure to disease, and little authority over the institutions that govern them. Heightened visibility refers to those moments when this underlying arrangement becomes harder to conceal, as in pandemics, aid shocks, or institutional withdrawals, to which the language of crisis is usually attached. The crisis form of global health asks us to begin with the former.

This historical pattern is inseparable from imperialism, colonialism, and racial capitalism. Few would disagree that international health emerged to protect European empires as opposed to a universal commitment to human flourishing [17]. Centering this history within a broader Global South retelling helps avoid dispossessing memory in ways that make global health look apolitical [18]. Colonizers developed health policies because disease threatened military occupation, labour productivity, administrative control, and the circulation of commodities and capital [17,19]. Malaria and yellow fever was important because they undermined the extraction of value from the tropics; cholera mattered because its spread triggered quarantines on shipping, interfering with trade and raised the cost of commercial exchange. The first International Sanitary Conference in 1855 emerged precisely from this problem, as Mediterranean states sought stricter quarantines (which Britain opposed) that would slow trade and increase costs [17]. Thus from the outset, international health was structured by a contradiction between disease control and accumulation. But the conventions did not resolve the contradiction; instead, they institutionalized it, and health became a way of managing circulation under imperialism [19].

The postwar period changed the grammar of global health but retained its logic. Creating WHO unified earlier regional health bodies and gave health a more universal institutional language, but this development was scaffolded by a Cold War struggle over world order [20]. The US and its allies supported multilateralism to stabilize a capitalist world in which newly independent states would not turn to the Soviet Union or embrace socialist development. Health aid and development policy were constitutive aspects of that strategy. Foreign aid expanded as a way of shaping political alignment and securing influence over the direction of state-building. Modernization theory gave this project an intellectual vocabulary, positing that all societies moved through universal stages of development and could be guided towards modernity through the right mix of capital, technology and institutions. Through this imaginary, colonizing states were framed as models of progress, obscuring the extractive relations that produced global inequality, and helped consolidate a technocratic politics in which health problems were solvable through targeted interventions while wider political economy remained intact.

Alma Ata briefly provided reprieve. Some newly independent states with their increasing majority and entry to the UN advocated health as a human right and for universal access to Primary Health Care, as part of a wider struggle against poverty and postcolonial dependency [21]. This movement culminated in the 1978 Alma-Ata Declaration on Primary Health Care; a landmark moment filled with optimism. Its rallying cry, “Health for All by the Year 2000,” proposed a shift away from narrowly focused, disease-specific programs toward inclusive, community-centered healthcare, linking health to broader postcolonial struggles against poverty and inequality [22]. But this vision was quickly sacrificed to neoliberal restructuring. Health systems were undermined by structural adjustment programs, and the comprehensive aims of Alma-Ata were abandoned in favor of more narrowly focused “selective primary care” [21]. These donor-friendly, vertical interventions were cheaper, more measurable, and easier to control. The WHO, under pressure from donors, scaled back its support for Alma-Ata, while its budget was increasingly reduced [23]. What was lost was a political understanding of health as crucial to sovereignty, redistribution and structural change.

The HIV/AIDS crisis in the 1980s and 1990s followed the same logic. Brandt argues that the crisis disrupted older, state-centered forms of international health, birthing global health and briefly opening space for rights language, activist intervention and demands for access to medicines [24,25]. New institutions like the Global Fund and PEPFAR emerged, channeling unprecedented funding to the epidemic, treatment expanded, and civil society gained visibility within global governance. But these gains did not transfer power in any fundamental sense. Donor states and philanthropic actors retained decision-making authority while recipient states remained subject to conditionality and external financing priorities [26]. Health systems in the Global South became increasingly oriented around AIDS at the expense of broader health needs [27]. AIDS responses were hailed as global solidarity but relations of care remained donor-driven, and crisis expanded control instead of dismantling it.

SARS, Ebola, and COVID-19 each repeated this structure with different emphases. SARS 2003 revived anxieties about global vulnerability and led to renewed surveillance capacities, through updates to the International Health Regulations (IHR), but enthusiasm for change faded once the threat receded [28]. The 2014–2016 Ebola crisis in West Africa exposed the violence of underfunded health systems in West Africa and made colonial afterlives difficult to ignore. Again, calls for reform surged, including proposals to strengthen WHO’s emergency response and to support the work of the Africa Centers for Disease Control [29]. As the crisis waned, momentum faded, and many reforms remained incomplete. COVID-19 has been the most dramatic case of all [30]. Predominantly white, wealthy countries hoarded vaccines, resisted patent waivers, and prioritized national recovery, while Black and brown people in the Global South were left to plead, wait and bury their dead [31]. Not even the vaccine inequity that animated the pandemic, and exposed structural problems, such as limited vaccine manufacturing in the Global South, weak health financing, and the legacy of extractive aid, could turn the tide [32].

## Discursive responses to U.S. Retrenchment

Like previous moments of crises, the 2025 US withdrawal from the WHO and suspension of most of its foreign aid funding, was narrated not as an opportunity to rethink the relations of global health, but as a moment to restore its hegemonic order. In the wave of commentaries catalyzed across publication houses, following the announcement from Washington D.C., commentators overwhelmingly treated the event as a geopolitical rupture—an abdication of leadership, a threat to multilateralism, and a moment requiring urgent institutional repair. But beneath their tonal variation, they held a shared assumption: that U.S., and Western leadership more broadly, is both virtuous and strategically indispensable. An inductive thematic analysis reveals five dominant narrative frames: leadership vacuum, geopolitical rivalry, EU stewardship, African self-reliance, and decentralized multilateralism (Table 1). While these framings gesture toward different futures, they largely operate within the lens of continuity.

**Table 1:** Discursive Framings of the 2025 U.S. Withdrawal from the WHO and Global Health Aid Cuts.

Discursive Frame	Narrative Summary	Underlying Assumptions	Marginalised Voices
Leadership Vacuum	U.S. withdrawal from WHO seen as destabilising; framed as a symbolic act causing disorder and systemic vulnerability [33,34,37–39,48,62].	U.S. leadership is essential for global health stability.	LMICs not seen as leaders, only recipients.
Geopolitical Rivalry	Fear of China or Russia filling the void; China depicted as undemocratic; global health framed as battleground for great powers [33,34,37,35,43,44].	Chinese influence is dangerous or illegitimate in global health.	Global South agency erased by great-power lens.
EU Leadership	EU urged to step in for strategic and humanitarian reasons; seen as opportunity for EU to reinforce leadership role [33,46–52].	EU must lead to prevent geopolitical and development collapse.	African agency instrumentalised in EU strategy.
African Sovereignty and Development	U.S. aid cuts framed by African voices as a chance to assert sovereignty, mobilise resources, and reject donor dependency [44,53–57,59–61,63].	Aid is a form of neocolonialism; sovereignty is developmental justice.	Risk of elite capture, managerialism, or external dependence.
Decentralised Multilateralism	Calls for reform of global governance via shared responsibility; WHO reform proposed; regional leadership promoted [33,39,43,62].	Global cooperation requires plural leadership; avoid hegemonic power.	Unclear operational mechanisms; risk of political dilution.

We found these frames from a targeted review of 23 English-language texts published between 1 January and 15 April 2025 that sought to understand how scholars, experts, and other stakeholders, understood the governance challenges aid cuts and WHO withdrawal presented and potential solutions. The initial step was a PubMed and Scopus search, which generated 728 entries; only three met our criteria after abstract screening. We located additional sources through targeted Google queries, resulting in a final total set of 23 publications. We have attached an annex with full inclusion and exclusion criteria.

## Leadership

A dominant frame, particularly among Global North commentators, casts the U.S. exit from the WHO as creating a perilous “leadership” or “political power vacuum” [33,34]. The U.S. exist was described as creating systemic instability, threatening WHO reform, and weakening global health security [33–37]. The WHO itself expressed deep regret, emphasizing that U.S. participation had helped catalyze the “largest set of [WHO] reforms in its history,” and warning that its departure would weaken global efforts to detect and respond to health emergencies [38]. References to a “void” carried the affective weight of crisis, implying not merely absence but imminent danger [39]. This framing positioned the U.S. not as one actor among many, but as the linchpin of global health governance. Its absence rendered as an existential threat. In doing so, these narratives re-centered the U.S. as necessary to the field’s coherence, effectively foreclosing any serious reckoning with its past or present role in undermining global health, for example, through policies like the Global Gag Rule, which restricts funding for NGOs that even mention reproductive justice [40]. Though formally limited to NGOs, the policy has caused widespread disruptions: clinics have closed, access to care has declined, and advocacy has been chilled [40–42].

## Geopolitics

Closely linked to this was the geopolitical rivalry frame, in which China and Russia’s growing influence was cast as a threat to Western values [37,43,44]. Commentators expressed concern that Beijing would use the vacuum to export authoritarian models, bypass multilateralism, and entrench undemocratic norms [37,39,43,45]. Others expressed concerns that civil society would be left exposed, emboldening “rivals” like China and Russia to deepen their influence in fragile states [46]. Yet these anxieties rarely interrogated the ideological content of Western values or U.S. leadership itself. Instead, they reinscribed Cold War binaries, treating the Global South not as an active subject but as terrain to be contested. In this frame, global health becomes the battleground for liberal versus illiberal governance models.

## European stewardship

A third narrative, foregrounding European stewardship, cast the EU as the natural heir to U.S. leadership. Commentators urged the EU to step up morally, financially, and institutionally, positioning it as the guarantor of multilateral stability [33,47–49]. This framing rested on the assumption that the preservation of global health depends on continuous leadership from the Global North. Some interventions presented this as an opportunity for the EU to project soft power, protect its economic interests, and secure geopolitical influence [50–52]. Others emphasized humanitarian obligations [53]. Yet both tendencies instrumentalized Global South health needs within a European strategic calculus.

## African sovereignty

In contrast, African commentators and regional actors offered a partial reframing. Here, the withdrawal was not simply a loss, but a provocation—a moment to confront long-standing dependencies. Some treated the crisis as a wake-up call, urging investment in local production, research autonomy, and regional integration [38,44,54]. This sovereignty frame presented aid as a neocolonial constraint and called for a break with donor-driven development [55,56]. Yet even these interventions were uneven. In some instances, self-reliance was reframed through a managerial lens: through efficiency, fiscal discipline, and institutional reform [57–60]. Others, more radically, gestured toward Pan-African political traditions and structural reorientation, asserting that “true development cannot be outsourced” [61]. Still, across much of the discourse, critiques of aid were decoupled from critiques of racial capitalism, limiting their transformative potential.

## Decentralized multilateralism

A final frame, decentralized multilateralism, proposed a shift away from hegemonic leadership altogether. Rather than replacing the U.S. with another dominant actor, some commentators advocated for shared governance structures that distribute authority more equitably across regions [39,62]. This included calls to empower institutions like the Africa CDC, diversify funding streams, and democratize decision-making within the WHO [33]. On the surface, this appears to challenge the existing order. But closer scrutiny reveals the limits of this vision. Proposals often lacked clarity on how power would be redistributed or how entrenched hierarchies would be dismantled. The language of decentralization remained technocratic, focused on efficiency and resilience, rather than structural transformation.

What unites these discursive frames is that none treats the crisis as produced by global health’s own governing relations. Rupture rhetoric is pervasive, but its political content is thin. Leadership is assumed; sovereignty is detached from redress; multilateralism is reduced to coordination rather than solidarity [33,34,37,39,62,64]. This is clearest in the silence around decoloniality. Despite years of debate on “decolonizing global health,” none of the texts engaged its substantive demands [65]. Even appeals to self-reliance remain severed from the histories of colonial subjugation and racialized dependency that made such dependency durable [53,54]. The result is a discourse that treats the withdrawal as a problem of institutional repair, not as an opening to confront the extractive relations of the system. In that discourse, the Global South appears mainly as the site where crisis lands, not as an author of what global health should become [33,38,63,66].

## The necessity of reparatory action in global health

Reparatory global health remains difficult for mainstream global health to imagine because the field has been shaped within a narrow political and epistemic horizon. It still privileges Euro-American assumptions about expertise and technical reason [67]. It treats inequality as a problem to be measured and mitigated without addressing the historical and structural relations that produced inequality [68]. The same narrowing appears institutionally. Funding streams, journals, policy, and multilateral institutions reward proposals that promise resilience and reform but are far less hospitable to arguments about restitution, debt cancellation, monopoly-breaking, or redistributing authority. Crisis discourse intensifies this problem because it creates urgency and then channels that urgency into rescue missions aimed at restoring governability and institutional continuity. The result is a field that hyper focuses on responding to acute events but not how to think about structural change. Reparatory global health, then, appears difficult to imagine not because it lacks intellectual substance, but because it requires a different way of seeing the problem: it requires us to treat colonialism, slavery, racial capitalism as constitutive formations of the present, and not historical episodes.

Reparations literature has already begun to do that work naming slavery, underdevelopment, inequality and climate vulnerability as part of a continuous historical present where unequal contribution to harm, unequal exposure, diminished adaptive capacity, and unjust enrichment are key considerations for redress [4–6,69,70]. Reparatory global health demands a break with the habits of thought that have made structural repair unintelligible. Contemporary African scholarship is helpful here because it links colonialism, economic inequality, and the need for structural justice in ways that help reproduce harm.

Reparatory global health rejects the premise that inequality can be managed through inclusion. The problem is not the underrepresentation of the Global South in existing institutions, but the very form that renders inclusion a substitute for power. Reform initiatives often seek to diversify actors without transforming the terms of participation. Yet when the field’s foundations are built on histories of dispossession, representation without redress becomes a mode of containment. Reparations, in this context, are not about settlement or apology. They are about structural realignment—redistributing authority over health priorities, research agendas, and funding flows [22,71–75]. Reparations must reconfigure the future, not only reconcile with the past on unjust terms [15].

Across the Global South, communities have long built infrastructures of health sovereignty, mutual aid, and collective living that have often flourished outside donor-led paradigms. Marginalized communities the world over have responded to systemic neglect not only with resilience but with innovation: from grassroots care networks to pan-African public health platforms, and indigenous knowledge systems to South-South cooperation. These are not peripheral stories; they are central to the making of global health. To draw from them is not to romanticize, but to recognize that alternatives already exist, and have for a long time. The question is not whether transformation is possible, but whether we are willing to shift our gaze and ground our imaginaries in these other inheritances. In place of crisis-as-loss, we offer crisis-as-opening: a moment not only to name what has failed, but to reclaim what has endured.

## Social movements

Reparatory global health is not a technical blueprint––it is a political struggle of which social movements is a central part. It is a paradigm shift: from managing populations to sustaining life, from crisis management to collective transformation [14,15]. Its horizon is not just dismantling systems of harm, but affirming life worlds grounded in solidarity and care. History provides both visions for imagining a world, otherwise, but it also cautions. Movements like Brazil’s Sanitary Reform, demands for a Global Social Protection Fund, the Black Panther Party’s community clinics, and Cuba’s medical brigades, have long modelled health as a site of resistance and repair [22,71–73,75,76]. These were not mere technical or policy interventions; they were confrontations with imperial power and assertions of health as a right bound to sovereignty and dignity, and as a key aspect of living in the world as a collective. Alma-Ata’s call for “Health for All” embodied this ethos before being gutted by structural adjustment and donor capture. Still, its emancipatory vision remains––a reminder that alternatives have always existed, displaced by those now offering reform. Reparatory global health takes its cues from these struggles. It insists that health is inseparable from land, labour, and liberation. And that justice cannot be integrated into global health as it stands, but the force that reconstitutes it.

## Legal forms

This requires confronting how global health has been shaped by legal forms. Global trade and intellectual property rules have been exposed as technologies of containment. From the TRIPS Agreement to donor-driven conditionalities, legal forms have protected property and external authority instead of guaranteeing access to essential medicines and strong health systems [77]. The COVID-19 vaccine apartheid underscored the need for a framework that enables poorer nations to produce essential medical goods for their populations. Acknowledging the ability of nations to manufacture, distribute, and innovate without relying on monopolies or external benevolence is an imperative. All countries should contribute, with wealthier nations bearing a larger share as part of their reparative responsibility, having extracted resources from the Global South, benefited, and failed to compensate. A reparative orientation would reframe law as a site of struggle, not stability. Crucially, this is not a call for more law but for law otherwise––attuned to historical theft and oriented toward collective flourishing.

## Epistemology

Equally critical is the terrain of epistemology. Who produces knowledge, whose expertise is validated, and which ontologies shape health interventions are all central to reparatory politics. Despite rhetorical commitments to “context” or “local ownership,” global health remains dominated by Euro-American biomedicine and evidence paradigms [78]. Reparatory global health insists on epistemic justice, not as a gesture of diversity, but as a challenge to colonial rationalities. It foregrounds Indigenous knowledge systems, relational models of care, and non-Western practices not as supplements to existing paradigms but as coequal foundations [16]. Sylvia Wynter’s call to reimagine the human beyond the colonial matrix becomes instructive here: the project is not to diversify the existing order, but to deconstruct it [79]. To claim reparations is to insist that knowledge itself has been racialized, and that healing must begin by unmaking its hierarchies. Otherwise, that is not truly “global health”.

## Institutional forms

This reconstitution must also engage with the form of global health institutions. The WHO, long constrained by donor earmarking and geopolitical pressure, operates within a relational form that privileges wealth over need, and vertical interventions over holistic care. A reparative approach would not merely increase funding or representation but would transform the institutional logic altogether. It would require binding obligations for equity, mechanisms for downward accountability, and the redistribution of agenda-setting power. Such a shift cannot be achieved through soft law or normative persuasion alone. It demands political struggle: to democratize governance, to rupture donor conditionalities, and to repurpose the system around justice rather than stability. Without institutional transformation, even the most progressive discourses will be absorbed and neutralized by the very relations they seek to challenge.

## One health

Reparatory global health must foreground ecological justice—not as an environmental add-on, but as foundational to collective health. The rise of One Health discourse, linking human, animal, and environmental well-being, offers a conceptual opening [80,81]. Yet in practice, it is often deployed in technocratic, surveillance-based terms, tracking zoonoses, anticipating outbreaks, protecting borders. It has been effectively co-opted from its long enduring roots in Indigenous knowledge systems across the world. A reparative lens would recast One Health as a political project: one that recognizes climate debt, land dispossession, and the role of racial capitalism in producing health crises. It would invest in agroecology, community-controlled food systems, and climate-resilient care infrastructure. Crucially, it would take seriously Indigenous ecological knowledge, not as data to be mined, but as forms of relations that unsettle Western ontologies.

## Conclusion

Our proposals are not final answers. What we offer is an invitation to rethink and reimagine how we live as a collective. What links them is a shared commitment to actual transformation, rather than applying superficial fixes to a system built on inequality. Strengthening regional leadership challenges the legacy of external control and paternalism. Ensuring rights to manufacture and access essential medicines targets the structural economic injustices that keep care out of reach for millions. And reimagining health through an ecological and reparative lens expands our vision to include long-ignored dimensions of justice: social, environmental, and epistemic.

None of this will be easy. These ideas confront entrenched colonial relations of power built on and sustained by extraction, accumulation, and dispossession, and will be resisted by those who profit from the status quo. But if global health is—as many now say—*burning*, then tinkering at the margins will not put out the fire. We need a different approach entirely. Reparatory work offers that shift towards abolition: it asks us to stop patching up a crumbling system and start addressing why it is failing in the first place. It invites us to rebuild a new, using materials that are just, inclusive, and designed to prevent future collapse. In moments of crisis, it is easy to think justice can wait. But it is precisely now, amid the wreckage, that we must prioritize it. Reparative justice is not a distraction; it is the extinguisher, and the blueprint, for something better. Only by rooting global health in solidarity, repair, and shared responsibility can we transform this endless cycle of crisis into a chance for collective well-being. This is the path to a future where global health is not shaped by domination or charity, but by mutual care, equity, and the collective pursuit of well-being for all.

## References

[1] Sheldrick M. Foreign Aid Is Shrinking—What Happens Next? Forbes. (accessed March 21, 2025). https://www.forbes.com/sites/globalcitizen/2025/02/25/foreign-aid-is-shrinking-what-happens-next/

[2] Aid cuts by the West threaten a tragic spike in AIDS-related deaths, new infections and drug resistance worldwide. Politics and International Studies. 2025 (accessed June 26, 2025). published online June 20. https://warwick.ac.uk/fac/soc/pais/research/centres/wicid/wicidblog/?newsItem=8ac672c49784040301978de3ae731eda

[3] Richardson ET, Keshavjee SA, Klinsky S, Chitre S, Matache M, Thorat A, et al. Reparations and distributive justice in global health. BMJ Glob Health. 2026;11(Suppl 1):e023078. doi: 10.1136/bmjgh-2025-023078 41871842 PMC13034365

[4] Hickel J, Keshavjee S, Burkett M, Richardson ET. Structural adjustment: damages, reparations and pathways to non-recurrence. BMJ Glob Health. 2026;11(Suppl 1):e017221. doi: 10.1136/bmjgh-2024-017221 41871845 PMC13034243

[5] Beckles H, Chitre S, Richardson ET. Reparations for Caribbean slavery and the potential impact on human well-being. BMJ Glob Health. 2026;11(Suppl 1):e017217. doi: 10.1136/bmjgh-2024-017217 41871846 PMC13034376

[6] Mullen AK, Richardson ET, Bassett MT, Darity WA. A plan for black American reparations. BMJ Glob Health. 2026;11(Suppl 1):e017216. doi: 10.1136/bmjgh-2024-017216 41871844 PMC13034248

[7] Gozzi N, Chinazzi M, Dean NE, Longini IM Jr, Halloran ME, Perra N, et al. Estimating the impact of COVID-19 vaccine inequities: a modeling study. Nat Commun. 2023;14(1):3272. doi: 10.1038/s41467-023-39098-w 37277329 PMC10241610

[8] Thambisetty S, McMahon A, McDonagh L, Kang HY, Dutfield G. Addressing vaccine inequity during the COVID-19 pandemic: The TRIPS intellectual property waiver proposal and beyond. CLJ. 2022;81:384–416.

[9] Ncube CB. Intellectual property norms in the polycrisis—(still) omnipresent, distracting, irrelevant? JIPLP. 2024;19(9):717–24. doi: 10.1093/jiplp/jpae054

[10] Vanni A. Patent games in the global south: pharmaceutical patent law making in Brazil, India and Nigeria. Oxford; New York: Hart; 2019.

[11] Petryna A. When experiments travel: clinical trials and the global search for human subjects. Princeton University Press; 2009.

[12] Sirleaf M. Health as global health. White. 2023;117:88–93.

[13] Knox R, Whyte D. Vaccinating capitalism: racialised value in the COVID-19 economy. Mortality. 2023;28(2):329–45. doi: 10.1080/13576275.2023.2169116

[14] Gilmore RW, Bhandar B, Toscano A. Abolition geography: essays towards liberation. Paperback ed. London New York: Verso; 2023.

[15] Táíwò OO. Reconsidering reparations. 1st ed. New York: Oxford University Press; 2022. doi: 10.1093/oso/9780197508893.001.0001

[16] Hodges S. The global menace. Soc Hist Med. 2012;25:719–28. 26345469 10.1093/shm/hkr166 PMC4558944

[17] Wenham C, Busby JW, Youde J, Herten-Crabb A. From imperialism to the “golden age” to the great lockdown: The politics of global health governance. Annu Rev Polit Sci. 2023;26:431–50.

[18] Manji F, Fletcher B. Claim no easy victories: The legacy of Amílcar Cabral. Philadelphia: Common Notions; 2024.

[19] Huber V. The unification of the global by disease? The international sanitary conferences on cholera, 1851–1894. Hist J. 2006;49:453–76.

[20] Bump JB. Global health and its limitations: an historical perspective. Health Syst Reform. 2025;11:2478681. 40202995 10.1080/23288604.2025.2478681

[21] Cueto M. The ORIGINS of primary health care and SELECTIVE primary health care. Am J Public Health. 2004;94(11):1864–74. doi: 10.2105/ajph.94.11.1864 15514221 PMC1448553

[22] Birn A-E, Krementsov N. “Socialising” primary care? The Soviet Union, WHO and the 1978 Alma-Ata Conference. BMJ Glob Health. 2018;3(Suppl 3):e000992. doi: 10.1136/bmjgh-2018-000992 30498594 PMC6242026

[23] Chorev N. The World Health Organization between north and south. Ithaca (NY): Cornell University Press; 2012.

[24] Brandt AM. How AIDS invented global health. N Engl J Med. 2013;368:2149–52. 23738542 10.1056/NEJMp1305297

[25] Jun 06, 2013. HIV Epidemic Brought About ‘Global Health,’ Serves As Model For Response To Other Diseases. KFF. (accessed May 23, 2025). https://www.kff.org/news-summary/hiv-epidemic-brought-about-global-health-serves-as-model-for-response-to-other-diseases/

[26] Haastrup T, Sekalala S. What have rights got to do with it? Evaluating ‘human rights’ as a practice within the Global Fund. Glob Health Governance. 2018;12:75–86.

[27] de Waal A. HIV/AIDS and the challenges of security and conflict. Lancet. 2010;375(9708):22–3. doi: 10.1016/S0140-6736(09)62175-9 20109848

[28] Fidler DP. From International Sanitary Conventions to Global Health Security: The New International Health Regulations. Chin J Int Law. 2005;4:325–92.

[29] Sambo U, Sule B. The 2014–2016 Ebola outbreak and responses of Africa. The Palgrave Handbook of Global Social Problems. Cham: Springer International Publishing; 2021. p. 1–19.

[30] The Independent Panel for Pandemic Preparedness & Response. COVID-19: Make it the Last Pandemic. 2021 (accessed June 25, 2023).https://theindependentpanel.org/wp-content/uploads/2021/05/COVID-19-Make-it-the-Last-Pandemic_final.pdf

[31] Prasad S, Shahid A, Co ELF, Khatri G, Cheema HA, Rocha ICN, et al. Vaccine apartheid: the separation of the world’s poorest and most vulnerable and the birth of Omicron. Ther Adv Vaccines Immunother. 2022;10:25151355221107975. doi: 10.1177/25151355221107975 35832726 PMC9272166

[32] Wenham C, Eccleston-Turner M, Voss M. The futility of the pandemic treaty: caught between globalism and statism. Int Aff. 2022;98(3):837–52. doi: 10.1093/ia/iiac023

[33] Kickbusch I. US exit from WHO: it is about much more than WHO. Lancet. 2025;405:444–6. 39884296 10.1016/S0140-6736(25)00163-1

[34] What a US withdrawal from WHO will mean for global health | Lowy Institute. (accessed May 24, 2025). https://www.lowyinstitute.org/the-interpreter/what-us-withdrawal-who-will-mean-global-health

[35] Hibbert CM. What the US exit from the WHO means for global health and pandemic preparedness. Northeastern Global News. 101 AD. (accessed May 24, 2025). https://news.northeastern.edu/2025/01/23/us-withdrawn-from-who/

[36] WHO chief asks countries to push Washington to reconsider its withdrawal. AP News. 2025 (accessed May 24, 2025). published online Feb 3. https://apnews.com/article/who-trump-tedros-global-health-a2eafc341cd2200e8800a2421d30bdfc

[37] Gostin LO, Meier BM, Pace L. A World Without WHO-A Crossroads for US Global Health Leadership. JAMA. 2025;333(12):1028–9. doi: 10.1001/jama.2024.28827 39823211

[38] Trump’s WHO withdrawal will be felt by the poorest. SciDev.Net. (accessed May 23, 2025). https://www.scidev.net/global/news/trumps-who-withdrawal-will-be-felt-by-the-poorest/

[39] Buse K, Gostin L, Kamarulzaman A, McKee M. The US withdrawal from the WHO: a global health crisis in the making. BMJ. 2025;388:r116. doi: 10.1136/bmj.r116 39837635

[40] Mavodza C, Goldman R, Cooper B. The impacts of the global gag rule on global health: a scoping review. Glob Health Res Policy. 2019;4:26. doi: 10.1186/s41256-019-0113-3 31485484 PMC6714436

[41] Skuster P, Sully EA, Friedrich-Karnik A. Evidence for ending the global gag rule: A multiyear study in two countries. 2024. published online April 16. 10.1363/2024.300502

[42] Rodgers Y. The global gag rule and women’s reproductive health: rhetoric versus reality. New York (NY): Oxford University Press; 2018.

[43] U.S. WHO Exit Could Expand China’s Influence. Think Global Health. 2025 (accessed May 23, 2025). published online Jan 30. https://www.thinkglobalhealth.org/article/us-who-exit-could-expand-chinas-influence

[44] Seifu B. Impacts of Foreign Assistance Cuts on Africa: USAID’s Role and Challenges Ahead. Modern Diplomacy. 2024 (accessed June 8, 2025). published online July 11. https://moderndiplomacy.eu/2024/07/11/impacts-of-foreign-assistance-cuts-on-africa-usaids-role-and-challenges-ahead/

[45] Güell O. La salida de EE UU de la OMS debilita la salud global y deja espacio a China para ganar influencia. El País. 2025 (accessed July 10, 2025). published online Jan 21. https://elpais.com/sociedad/2025-01-21/la-salida-de-ee-uu-de-la-oms-debilita-la-salud-global-y-deja-espacio-a-china-para-ganar-influencia.html

[46] Europe must step up to mitigate US aid suspension. Renew Europe. 2025 (accessed June 8, 2025). published online Feb 12. https://www.reneweuropegroup.eu/news/2025-02-12/europe-must-step-up-to-mitigate-us-aid-suspension

[47] US aid cuts leave EU member hopefuls in the lurch. The Parliament Magazine. 2025 (accessed June 9, 2025). published online March 13. https://www.theparliamentmagazine.eu/news/article/us-aid-cuts-leave-eu-member-hopefuls-in-the-lurch

[48] Broeck EVD. With the US leaving the WHO, Europe must urgently increase its global leadership in health preparedness – not weaken it. CEPS. 2025 (accessed May 23, 2025). published online March 13. https://www.ceps.eu/with-the-us-leaving-the-who-europe-must-urgently-increase-its-global-leadership-in-health-preparedness-not-weaken-it/

[49] Piouff CL. Filling the void: Why the EU should step up amid Trump’s foreign aid cuts. ECFR. 2025 (accessed June 8, 2025). published online Jan 24. https://ecfr.eu/article/filling-the-void-why-the-eu-should-step-up-amid-trumps-foreign-aid-cuts/

[50] Global health chiefs urge EU to step up amid US funding cuts. POLITICO. 2025 (accessed June 8, 2025). published online March 28. https://www.politico.eu/article/global-health-chiefs-urge-eu-to-step-up-amid-us-funding-cuts/

[51] O’Connell S, March 2025 DK// 19. Opinion: Aid cuts in US, UK, and beyond mean EU institutions must step up. Devex. 2025 (accessed June 8, 2025). published online March 19. https://www.devex.com/news/sponsored/opinion-aid-cuts-in-us-uk-and-beyond-mean-eu-institutions-must-step-up-109656

[52] Giusto H. How Europe can step up on trade and aid as the US retreats from global institutions. Foundation for European Progressive Studies. 2025 (accessed June 8, 2025). published online April 11. https://feps-europe.eu/how-europe-can-step-up-on-trade-and-aid-as-the-us-retreats-from-global-institutions/

[53] How do USAid cuts impact Africa? - L’Osservatore Romano. (accessed June 8, 2025). https://www.osservatoreromano.va/en/news/2025-04/ing-004/how-do-usaid-cuts-impact-africa.html

[54] Chimtom/Crux NK. Africa must address aid dependancy due to US policy shift under Trump - Catholic Herald. 2025 (accessed June 8, 2025). published online March 7. https://thecatholicherald.com/shift-in-us-policy-means-africa-must-look-at-disease-of-aid-dependancy/

[55] Eyaaz. No minerals for the US if aid is cut, Gwede Mantashe threatens. The Mail & Guardian. 2025 (accessed June 8, 2025). published online Feb 3. https://mg.co.za/politics/2025-02-03-no-minerals-for-the-us-if-aid-is-cut-gwede-mantashe-threatens/

[56] Why some Africans see opportunity in foreign-aid cuts. The Economist. (accessed June 8, 2025). https://www.economist.com/middle-east-and-africa/2025/03/06/why-some-africans-see-opportunity-in-foreign-aid-cuts

[57] Trump’s funding cuts will hurt South Africa and the region. ISS Africa. (accessed June 8, 2025). https://issafrica.org/iss-today/trump-s-funding-cuts-will-hurt-south-africa-and-the-region

[58] Mahjar-Barducci A. African Intellectuals On U.S. Foreign Aid Pause: ‘Time To Break Free From Foreign Aid Dependency’; ‘A Challenge And An Opportunity’; ‘Relying On Aid From The West Limits Our Opportunities To Be Industrious And Creative’. The Middle East Media Research Institute. 2025 (accessed June 10, 2025). published online Feb 18. https://www.memri.org/reports/african-intellectuals-us-foreign-aid-pause-time-break-free-foreign-aid-dependency-challenge#_edn4

[59] Mahjar-Barducci A. Foreign Aid Has Perpetuated Dictatorships. The Middle East Media Research Institute. 2025 (accessed June 10, 2025). published online Jan 30. https://www.memri.org/reports/foreign-aid-has-perpetuated-dictatorships

[60] Desk TKT-E. When US Aid Shrinks, Execution Must Rise to Bridge Gap. 2025 (accessed June 8, 2025). published online June 3. https://thekenyatimes.com/politics/when-us-aid-shrinks-execution-must-rise/

[61] Nicholas Okumu: USAID funding cuts expose a truth Africa must accept. The Star. (accessed June 8, 2025). https://www.the-star.co.ke/opinion/2025-02-18-nicholas-okumu-usaid-funding-cuts-expose-a-truth-africa-must-accept

[62] Buse K, Pagel C, Kamarulzaman A, McKee M. President Trump wants an alternative to the World Health Organization: how should we respond? BMJ. 2025;388:r188. doi: 10.1136/bmj.r188 39884701

[63] Adeyinka DA, Ologunagba B, Olakunde BO. US funding cuts as a catalyst for African-led HIV solutions. Lancet HIV. 2025;12(4):e248–9. doi: 10.1016/S2352-3018(25)00049-9 40031936

[64] The Providence Journal Subscription Offers, Specials, and Discounts. (accessed May 23, 2025). https://subscribe.providencejournal.com/restricted

[65] Krugman DW. Global health and the elite capture of decolonization: On reformism and the possibilities of alternate paths. PLOS Glob Public Health. 2023;3(6):e0002103. doi: 10.1371/journal.pgph.0002103 37384634 PMC10309605

[66] Navigating US global health aid cuts: What can past donor exits teach us? Brookings. (accessed April 9, 2025). https://www.brookings.edu/articles/navigating-us-global-health-aid-cuts-what-can-past-donor-exits-teach-us/

[67] Affun-Adegbulu C, Adegbulu O. Decolonising Global (Public) Health: from Western universalism to Global pluriversalities. BMJ Glob Health. 2020;5(8):e002947. doi: 10.1136/bmjgh-2020-002947 32819916 PMC7443258

[68] Rad J. Health inequities: a persistent global challenge from past to future. Int J Equity Health. 2025;24(1):148. doi: 10.1186/s12939-025-02526-y 40410748 PMC12103002

[69] Klinsky S, Chitre S, Richardson ET, Burkett M. Climate reparations for threats to health. BMJ Glob Health. 2026;11(Suppl 1):e017222. doi: 10.1136/bmjgh-2024-017222 41871847 PMC13034377

[70] Gilmore S, Sandoval-Villalba C. From harm to healing: transforming the lives of victims of conflict-related sexual violence through reparation. BMJ Glob Health. 2026;11(Suppl 1):e017220. doi: 10.1136/bmjgh-2024-017220 41871843 PMC13034339

[71] Fleck F. Consensus during the Cold War: back to Alma-Ata. Bull World Health Organ. 2008;86(10):745–6. doi: 10.2471/blt.08.031008 18949208 PMC2649520

[72] Bassett MT. Beyond Berets: The Black Panthers as Health Activists. Am J Public Health. 2016;106(10):1741–3. doi: 10.2105/AJPH.2016.303412 27626339 PMC5024403

[73] Bassett MT. No Justice, No Health: the Black Panther Party’s Fight for Health in Boston and Beyond. J Afr Am St. 2019;23(4):352–63. doi: 10.1007/s12111-019-09450-w

[74] Brown TM. Working With the Panthers to Transform Health Care for Poor Black Communities. Am J Public Health. 2016;106(10):1756–7. doi: 10.2105/AJPH.2016.303402 27552281 PMC5024398

[75] Nelson A. Body and soul: the Black Panther party and the fight against medical discrimination. Minneapolis: University of Minnesota Press; 2011.

[76] Shekighenda L. Tanzania: Nyerere’s Indelible Impact On Tanzania’s Health Sector. Tanzania Daily News. 2024 (accessed May 24, 2025). published online Oct 14. https://allafrica.com/stories/202410140084.html

[77] Sekalala S. Ending pandemics within the shadow of trade: reconciling equity in global health with coloniality. Camb Int Law J. 2025;14(1):4–29. doi: 10.4337/cilj.2025.01.01 41567276 PMC7618657

[78] Abimbola S. The foreign gaze: essays on global health. Montpellier: IRD Editions; 2024.

[79] Wynter S. Unsettling the coloniality of being/power/truth/freedom: Towards the human, after man, its overrepresentation--an argument. ncr. 2003;3:257–337.

[80] Baquero OS, Benavidez Fernández MN, Acero Aguilar M. From Modern Planetary Health to Decolonial Promotion of One Health of Peripheries. Front Public Health. 2021;9:637897. doi: 10.3389/fpubh.2021.637897 34178913 PMC8222522

[81] Kamenshchikova A, Wolffs PFG, Hoebe CJPA, Horstman K. Anthropocentric framings of One Health: an analysis of international antimicrobial resistance policy documents. Crit Public Health. 2021;31(3):306–15. doi: 10.1080/09581596.2019.1684442

